# Spatial distribution of malaria problem in three regions of Ethiopia

**DOI:** 10.1186/1475-2875-12-207

**Published:** 2013-06-17

**Authors:** Dawit G Ayele, Temesgen T Zewotir, Henry G Mwambi

**Affiliations:** 1 School of Mathematics, Statistics and Computer Science, University of KwaZulu-Natal, Pietermaritzburg, Private Bag X01, Scottsville 3209, South Africa

**Keywords:** Mixed model, Rapid diagnostic test, Spatial statistics, Variogram, Kriging

## Abstract

**Background:**

The transmission of malaria is the leading public health problem in Ethiopia. From the total area of Ethiopia, more than 75% is malarious. The aim of this study was to identify socio-economic, geographic and demographic risk factors of malaria based on the rapid diagnosis test (RDT) survey results and produce the prevalence map of the area illustrating variation in malaria risk.

**Methods:**

This study accounts for spatial correlation in assessing the effects of socio- economic, demographic and geographic factors on the prevalence of malaria in Ethiopia. A total of 224 clusters of about 25 households each were selected from the Amhara, Oromiya and Southern Nation Nationalities and People’s (SNNP) regions of Ethiopia. A generalized linear mixed model with spatial covariance structure was used to analyse the data where the response variable was the presence or absence of malaria using the RDT.

**Results:**

The results showed that households in the SNNP region were found to be at more risk than Amhara and Oromiya regions. Moreover, households which have toilet facilities clean drinking water, and a greater number of rooms and mosquito nets in the rooms, have less chance of having household members testing positive for RDT. Moreover, from this study, it can be suggested that incorporating spatial variability is necessary for understanding and devising the most appropriate strategies to reduce the risk of malaria.

## Background

Malaria is a life-threatening disease affecting the world’s most under-developed countries and regions where basic healthcare infrastructure is lacking [1] as well some developed countries. Malaria is a major cause of morbidity and mortality in Africa, especially in sub-Saharan African countries [1]. It is a leading cause of death amongst children in many African countries [2]. With 68% of the total population of Ethiopia living in areas at risk of malaria [3], it is a major public health problem and for many years the prime cause of illness and death [3,4]. From the total population of Ethiopia (77,127,000 in 2007), more than 50 million people are at risk from malaria [5]. In general, 4–5 million people are affected by malaria annually [6,7].

Epidemics of malaria are relatively frequent [8,9] involving highland or highland fringe areas of Ethiopia, mainly areas 1,000-2,000 meters above sea level [10-12]. Notably this altitude covers 48% of the regions of Amhara, Oromiya and Southern Nations Nationalities and People’s regions of Ethiopia. Malaria epidemics have serious consequences for Ethiopia’s subsistence economy as the malaria transmission peaks during the major harvesting seasons. To control the risk of malaria, early diagnosis and prompt treatment is one of the key strategies. To diagnose malaria, clinical diagnosis is the most widely used. But, laboratory facilities are not available in all areas of the country [13,14]. The standard method to diagnose malaria is microscopy. However, this form of diagnosis is not accessible or affordable in most peripheral health facilities. The recent introduction of rapid diagnostic tests (RDT) for malaria is a significant step forward in case detection, timely treatment and management, and reduction of unnecessary treatment. RDT could be used in malaria diagnosis during population-based surveys and to provide immediate treatment based on the results.

RDTs offer the potential to extend accurate malaria diagnosis to areas where microscopy services are not available such as in remote locations or after regular laboratory hours. Rapid malaria diagnostic tests have been developed in the lateral flow format [15]. These tests use finger-stick or venous blood, which takes only 10 to 15 minutes to complete, and do not require a laboratory. Non-clinical staff can easily learn to perform the test and interpret the results [16].

It is essential to identify the socio-economic and demographic risk factors associated with the prevalence of malaria using data obtained from the rapid diagnosis test. Such a study of the identification of the socio-economic and demographic risk factors is helpful in identifying households who have a critical need for intervention. In previous studies, Ayele, Zewotir and Mwambi (2012) have concluded that malaria problem in Amahara, Oromiya and SNNP regions of Ethiopia are associated with key socio-economic, demographic and geographic factors, in particular it was noted that poverty levels of households are highly associated with the risk of malaria. Nevertheless the spatial distribution of malaria was not considered or investigated [17]. Though identification of the household characteristics is essential for grass root level intervention, the government goals and targets are focused on achieving malaria eradication/reduction within specific geographical areas. Such studies are limited, and hence the conception of this study. Therefore, the objective of this study is to undertake a statistical analysis of malaria incidence. This will identify important socio-economic, demographic and geographic variables associated with the disease and ultimately a prevalence map of the area illustrating variations in malaria risk.

## Methods

### Study design

**Figure 1:**
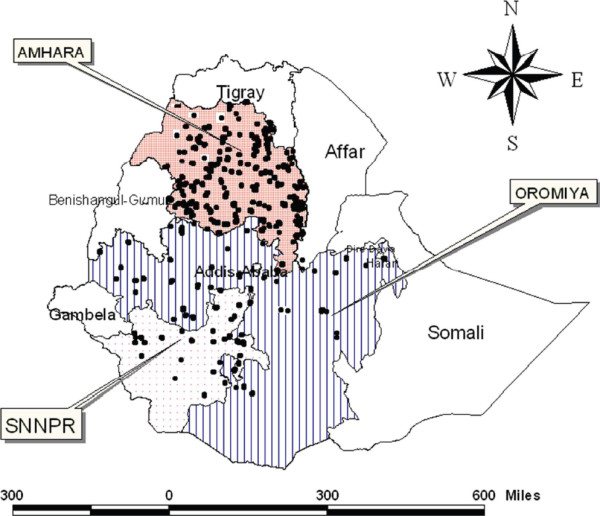
Map of Ethiopia showing the surveyed households.

From December 2006 and January 2007, a baseline household cluster malaria survey was conducted by The Carter Center (TCC). The questionnaire was developed as a modification of the Malaria Indicator Survey (MIS) Household Questionnaire. The questionnaire had two parts; the household interview and malaria parasite form. For this survey, the sampling frame in each of the rural populations of Amhara, Oromiya and SNNP regions was a *Kebele* (the smallest administrative unit in Ethiopia). The study area with the selected households is presented in Figure 1. From the three regions, 5,708 households located in 224 clusters were included in the survey. Out of these households, Amhara, Oromiya and SNNP regions covered 4,101 (71.85%), 809 (14.17%) and 798 (13.98%) households respectively. Prior to conducting the survey, 224 *Kebeles* were selected. From each *Kebele*, 12 households (even numbered households) were selected for malaria tests. In the survey each room in the house was listed separately. In addition to the number of rooms and number of nets, the persons sleeping under each net were listed. The detailed sampling procedure is presented in [17-19].

Before testing for malarial parasites, consent was obtained from the participants. To collect the sample, finger-prick blood was collected from the participants for the malaria rapid diagnostic test. The test used is known as *ParaScreen* which is capable of detecting both *Plasmodium falciparum* and other *Plasmodium* species. Participants with positive rapid tests were immediately offered treatment according to national guidelines.

### Variables of interest

#### Response variable

The outcome of interest is the malaria rapid diagnosis test (RDT) result. RDTs assist in the diagnosis of malaria by detecting evidence of malaria parasites in human blood and are an alternative to diagnosis based on clinical grounds or microscopy, particularly where good quality microscopy services cannot be readily provided. Thus, the response variable is binary, indicating whether or not a person is positive for malaria using the RDT.

#### Independent variables

The independent variables or covariates were the baseline socio-economic status, demographic and geographic variables including gender, age, family size, region, altitude, main source of drinking water, time taken to collect water, toilet facilities, availability of electricity, radio and television, total number of rooms, main material of the room’s walls, main material of the room’s roof, main material of the room’s floors, incidence of anti-mosquito spraying in the past 12 months, use of mosquito nets and total number of nets. Malaria test (RDT result), age and sex were collected at individual level. Altitude, main source of drinking water, time taken to collect water, toilet facilities, availability of electricity, radio, television, total number of rooms, main material of the room’s walls, main material of the room’s roof, main material of the room’s floor, use of anti- mosquito spray in the past 12 months, use of mosquito nets and total number of nets were all collected at household level.

### The statistical model

The distribution of malaria is nonrandom across a landscape in areas of higher or lower transmission intensity and malaria risk. The transmissions are separated by greater or lesser distances from each other. Based on geographical aggregation, there are two distinct levels. These are, the focal unit of malaria transmission, the area over which human malaria is actively transmitted originating from a specific aquatic breeding site and the household or other reasonably identified point of contact between a small group of humans and mosquito vectors. The baseline household cluster malaria survey which was conducted by The Carter Center from December 2006 to January 2007 includes the geographical locations of the reference for each household. Therefore, it is of interest to know whether the data display any spatial autocorrelation. Furthermore, it is important to check whether surveys that are near in space have malaria prevalence or incidence that is similar with the surveys that are far apart. This is important because spatially correlated data cannot be regarded as independent observations. If the analysis does not take account of the correlation structure of the data, the estimates obtained from the analysis may be inaccurate because of the underestimated standard errors. Therefore, the objective of this study is to undertake statistical analysis of malaria incidence to identify important socio-economic, demographic and geographic variables associated with the disease and to produce prevalence maps of the area illustrating the variation in malaria risk using spatial statistics analysis. Spatial statistics can be divided into three methods. These are: point pattern analysis, methods for lattice data and geostatistics [20,21]. *Point referenced data* is often called geocoded or geostatistical data. *Areal data* is often called lattice data. Some spatial data sets feature both point and areal-level data. *Point pattern data*: The response is often fixed (occurrence of the event), and only the locations where it occurs are thought of at random. Of these, the geostatistical approach is most relevant to epidemiological analysis which is conducted at the landscape scale and based on remote sensing [22-24].

A common approach to integrate spatially correlated data with the random effects and proceed with maximum likelihood based approaches for estimating the covariate and covariogram parameters, is based on the theory of generalized linear mixed models (GLMM). Using GLMM, numerical approximation can be implemented [20,25].

Non-Gaussian spatial problems may be formally analysed in the context of generalized linear mixed models (GLMM). Specification of the likelihood of the random variable *y*(*s*) is required where *s* generally denotes the location the observation is made. As in classical generalized linear models (GLMs), there is a canonical parameter corresponding to the distribution, which is nominally a function of the location parameter via the link function *g*(.) for the distribution. This function is assumed to be linear in the explanatory variables. In the classical formulation of GLMs containing only fixed effects, g(μ) = ***Xβ***, where **X** is the matrix of explanatory variables [26-30]. To incorporate a spatial process, we assume y(s_*i*_|*α*) is conditionally independent for any location *s*_*i*_ with conditional mean *E*[*y*(*s*_*i*_)|*α*] = *μ*(*s*_*i*_). The parameter α is used to define the distribution of *s*. Then, the spatially correlated random effect is incorporated into the linear predictor as:

(1) $$g\left(\mu \right)=X\beta +Z\alpha$$

where ***X*** and ***Z*** are the design matrix. The error term accommodates over-dispersion relative to the mean-variance relationship implied by the distribution under consideration. The random effect at location s_i_, *α* ∼ *Gau*(0, ∑ _*α*_(*θ*)) and $\epsilon ∼\mathrm{Gau}\left(0,\sigma_{\epsilon }^{2}I\right)$, with spatial correlation is parameterized by θ in ∑ _*α*_(*θ*) [20]. Note that *s*_*i*_ is just one location. *s* = (*s*_*l*_, ,*s*_*k*_)’ denotes a vector of *k* locations with variance-covariance matrix .

Spatial dependence may be represented by a range of functions [31]. To describe spatial correlation of observations, there are three major functions used in geostatistics. These major functions are the correlogram, the covariance, and the semivariogram. Semivariogram is also more simply called the variogram. In geostatistics, the variogram is the key function and is used to fit a model for the spatial correlation in the data. The model which is obtained using the variogram is used in kriging estimation procedures, a method which was first used in minimizing [23]. Moreover, variogram models are also used to understand maximum distances of spatial autocorrelation which can further be used in construction of search parameters for different interpolation techniques. A variogram represents both structural and random aspects of the data under consideration. The structural part of the variogram model is represented by the range of a variogram. Furthermore, the variogram values increase with increases in the distance of separation until it reaches the maximum at a distance known as the “range”. To develop the variogram, assume *μ*(s) is a constant, that is constant mean *μ*(s), and define

(2) $$\mathrm{var}\left\{Z\left(s_{1}\right)-Z\left(s_{2}\right)\right\}=2y\left(s_{1}-s_{2}\right)$$

In statement (2), the variance of *s*_*1*_ and *s*_*2*_ is through their difference *s*_*1*_-*s*_*2*_, and the process which satisfies this property is called intrinsically stationary. The function *2y*(.) is called the variogram and *y*(.) the semivariogram.

The other concept here is isotropy. Suppose the process is intrinsically stationary with semivariogram [*y*(*h*), *h* ∈ **R**^d^. If *y*(*h*) = *Y*_0_(|*h*|) for some function *Y*_*0*_, i.e. if the semivariogram depends on its vector argument *h* only through its length |*h*|, then the process is isotropic. Therefore, a process which is both intrinsically stationary and isotropic is also called homogeneous. Isotropic processes are convenient to deal with because there are a number of widely used parametric forms for *y*_*0*_(*h*). Using semivariance *y*_*0*_(*t*) for interval distance class *t*, lag distance interval *t*, *c*_*0*_ (nugget variance) ≥ 0,*c*_*1*_ (structural variance) ≥ *c*_*0*_ and *R* is the range parameter R, some of the examples are:

1. Spherical:

$$\begin{array}{ccc} & 0 & \mathrm{ift}=0,\\ y_{0}\left(t\right)= & \left\{c_{0}\right)+c_{1}t\left\{\frac{3}{2}\frac{t}{R}-\frac{1}{2}{\left(\frac{t}{R}\right)}^{3}\right\} & \mathrm{ift}<t\leq R,\\ & c_{0}+c_{1}t & \mathrm{ift}\geq R. \end{array}$$

It is a convenient form because it increases from a positive value c_0_ when *t* is small, levelling at the constant c_0_ + c_1_ at *t* = *R*. This is the so-called "nugget/range/ sill" form which is often considered a realistic and interpretable form for a semivariogram.

2. Exponential:

γ0t=0ift=0,c0+c11−e−tRift>1.

This is simpler in functional form than the spherical case (and valid for all d) but without the finite range of the spherical form. The parameter ***R*** has a similar interpretation to the spherical model however, of fixing the scale of variability.

3. Gaussian:

γ0t=0ift=0,c0+c11−e−t2R2ift>1.

4. Exponential-power form:

γ0t=0ift=0,c0+c11−e−tRpift>1.

Here *0* < *p* ≤ *2*. This form generalizes both the exponential and Gaussian forms, and forms the basis for the families of spatial covariance functions introduced by Sacks *et al*. in 1989 [32]. However, in generalizing the results from one dimension to higher dimensions, these authors used a product form of covariance function in preference to constructions based on isotropic processes [33].

### Spatial prediction

Modelling spatial data is not only useful for identifying significant covariates but for producing smooth maps of the outcome by predicting it at unsampled locations. Spatial prediction is usually referred to as kriging. Kriging is an optimal interpolation based on regression against observed values of surrounding data points, weighted according to spatial covariance values. Interpolation refers to an estimation of a variable at an unmeasured location from observed values at surrounding locations [34]. Kriging has some advantages. These advantages are that it

•helps to compensate for the effects of data clustering, assigning individual points within a cluster less weight than isolated data points,

•gives an estimate of estimation error (kriging variance), along with an estimate of the variable,

•ensures availability of estimation error which provides a basis for stochasticity,

•allows simulation of possible realization.

The spatial prediction which is called kriging can statistically be defined as follows.

Let ***Y***_0_ be a vector of the binary response at a new, unobserved location *s*_*0i*_, *i* = *l*, ,*n*_*0*_ . Following the maximum likelihood approach, the distribution of *Y*_0_ is given by

(3) $$P\left(Y_{0}|\hat{\beta },\hat{U},{\hat{\sigma }}^{2},\hat{ø}\right)=\int P\left(Y_{0}|\hat{\beta },U_{0}\right)P\left(U_{0}|\hat{U},{\hat{\sigma }}^{2},\hat{ø}\right)dU_{0}$$

Where $\hat{\beta },{\hat{\sigma }}^{2}$ and $\hat{\phi }$ are the maximum likelihood estimates of the corresponding parameters. As part of the iterative estimation process, for penalized quasi-likelihood (PQL), $\hat{U}$ can be derived [35]. $P\left(Y_{0}\left|\hat{\beta },U_{0}\right)\right)$ is the Bernoulli-likelihood at new locations and $P\left(U_{0}\left|\hat{U},{\hat{\sigma }}^{2},\hat{\phi }\right)\right)$ is the distribution of the spatial random effects *U*_*0*_ at new sites, given $\hat{U}$ at observed sites and is assumed to follow the normal distribution that is

(4) $$P\left(Y_{0}\left|\hat{U},{\hat{\sigma }}^{2},\hat{\phi }\right)\right)=N\left(\sum_{01}\sum_{11}^{1}\hat{U},\sum_{00}-\sum_{01}\sum_{11}^{1}\sum_{10}\right)$$

With *Σ*_*11*_ = E(UU’), Σ_*00*_ = *E*(*U*_*o*_*U*’_*0*_) and $\sum_{01}=\sum_{01}^{t}=E\left(U_{o}U{'}_{0}\right)$. The mean of the Gaussian distribution in (4) is the classical kriging estimator [20].

The Bayesian predictive distribution of ***Y***_*0*_ is given by

(5) $$\begin{array}{l} P\left(Y_{0}\left|Y\right)\right)=\int P\left(Y_{0}\left|\beta \right),U_{0})P\left(U_{0},\left|U\right),\sigma^{2},\phi \right)\mathrm{xP}\left(\beta ,U,\sigma^{2},\phi \left|Y\right)\right)\right)\\ \;\times \mathrm{d\beta d}U_{0}\mathrm{dUd}\sigma^{2}\mathrm{d\phi} \end{array}$$

Where *P*(*β*, *U*, *σ*^2^, *ϕ*|*Y*) is the posterior distribution of the parameters obtained by the Gibbs sampler or the sampling importance re-sampling (SIR) approach. Simulation-based Bayesian spatial prediction is performed by consecutive draws of samples from the posterior distribution, the distribution of the spatial random effects at new locations and the Bernoulli-distributed predicted outcome. The maximum likelihood predictor (3) can be viewed or interpreted as the Bayesian predictor (5), with parameters fixed at their maximum-likelihood estimates. In contrast to Bayesian kriging, classical kriging does not account for uncertainty in estimation of *β* and the covariance parameters.

The data was analysed by fitting a generalized linear mixed model (GLMM) using SAS 9.2 PROC GLIMMIX.

### Analysis and results

Using the identified thirteen main effects and six two-way and three-way interaction effects [17] several covariance structures including SP(EXP) (Exponential), SP(EXPA) (Anisotropic Exponential), SP(EXPGA)( (2D Exponential, Geometrically Anisotropic), SP(GAU) (Gaussian), SP(GAUGA)( (2D Gaussian), (Geometrically Anisotropic), SP(LIN) (Linear), SP(LINL) (Linear Log), SP(MATERN)(Matérn), SP(MATHSW)(Matérn (Handcock-Stein-Wallis)), SP(POW) (Power), SP(POWA) (Anisotropic Power), SP(SPH) (Spherical) and SP(SPHGA)( (2D Spherical, Geometrically Anisotropic) were fitted but SP(GAU) (Gaussian) was found to be the best spatial covariance structure for the model [36].

**Figure 2:**
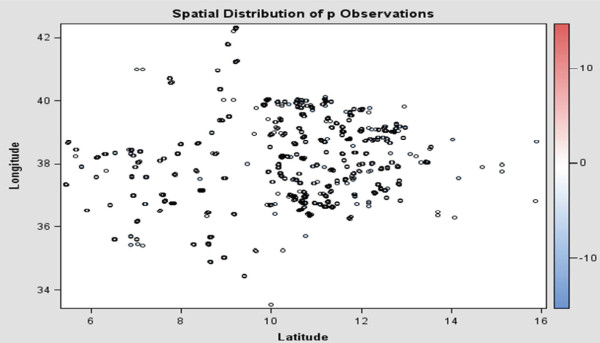
Scatter plot for the malaria prevalence.

**Figure 3:**
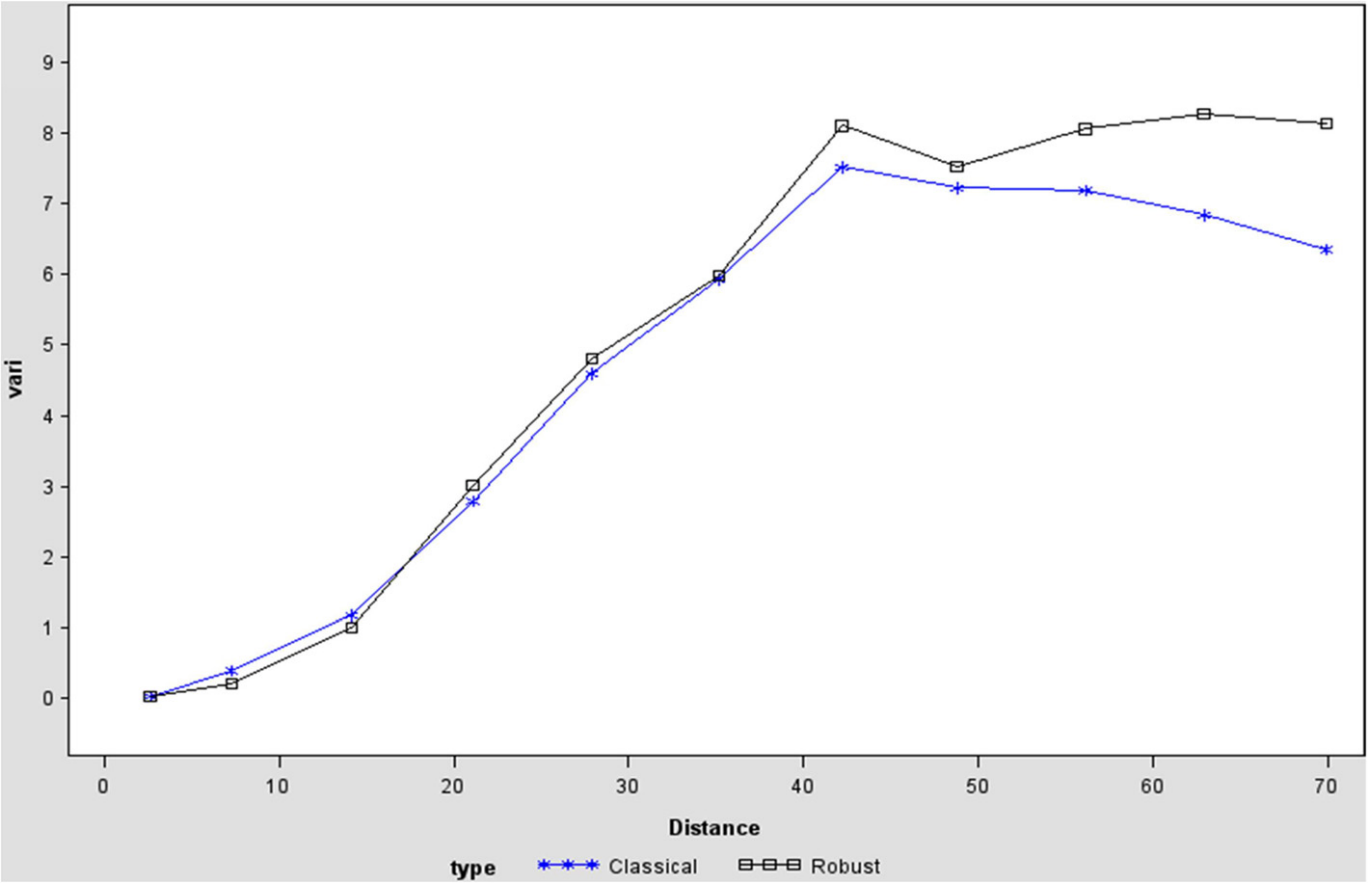
Distribution of observed malaria infected households.

**Figure 4:**
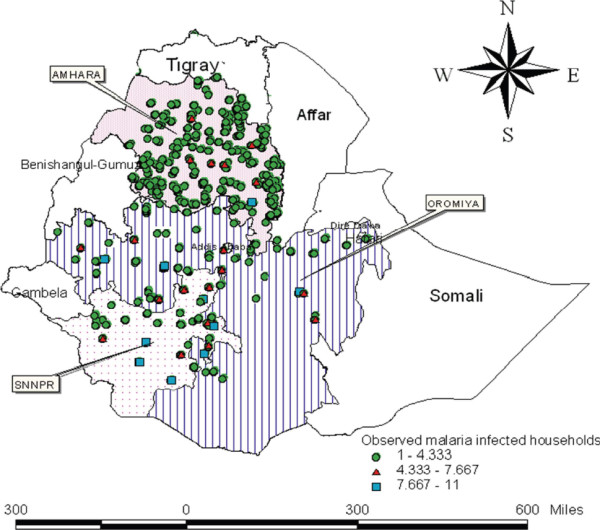
Distribution of observed malaria rapid diagnosis test.

The result presented in Figure 2 is a spatial scatter plot of the observed data. The scatter plot suggests distribution which is not indicative of a uniformly spread of the RDT measurements throughout the prediction area. No direct inference can be made about the existence of a surface trend in the data. However, the apparent stratification of RDT values might indicate a nonrandom trend. The Spatial Autocorrelation is an inferential statistic tool, which is important to test for randomness. This means that the results of the analysis are always interpreted within the context of its null hypothesis of a random occurrence of events. For the randomness test Moran’s and Geary's C tests can be used [37-41]. Furthermore, the distribution of observed malaria infected households and distribution of observed malaria rapid diagnosis test is presented in Figures 3 and 4.

For these tests, the null hypothesis states that the spatial distribution of feature values is the result of random spatial processes. The result from Moran’s (Z value = −40.4 and p – value < .0001) and Geary's c (Z value = −11.2 and P-value < .0001) tests indicate that the spatial distribution of feature values is not the result of random spatial processes. The Z values are negative for both Moran’s and Geary’s C tests. This indicates that the spatial distribution of high values and low values in the dataset is more spatially dispersed than would be expected if underlying spatial processes were random. A dispersed spatial pattern often reflects some type of competitive process, i.e., a feature with a high value repels other features with high values; similarly, a feature with a low value repels other features with low values. The observed spatial pattern of feature values could not very well be one of many possible versions of complete spatial randomness.

**Figure 5:**
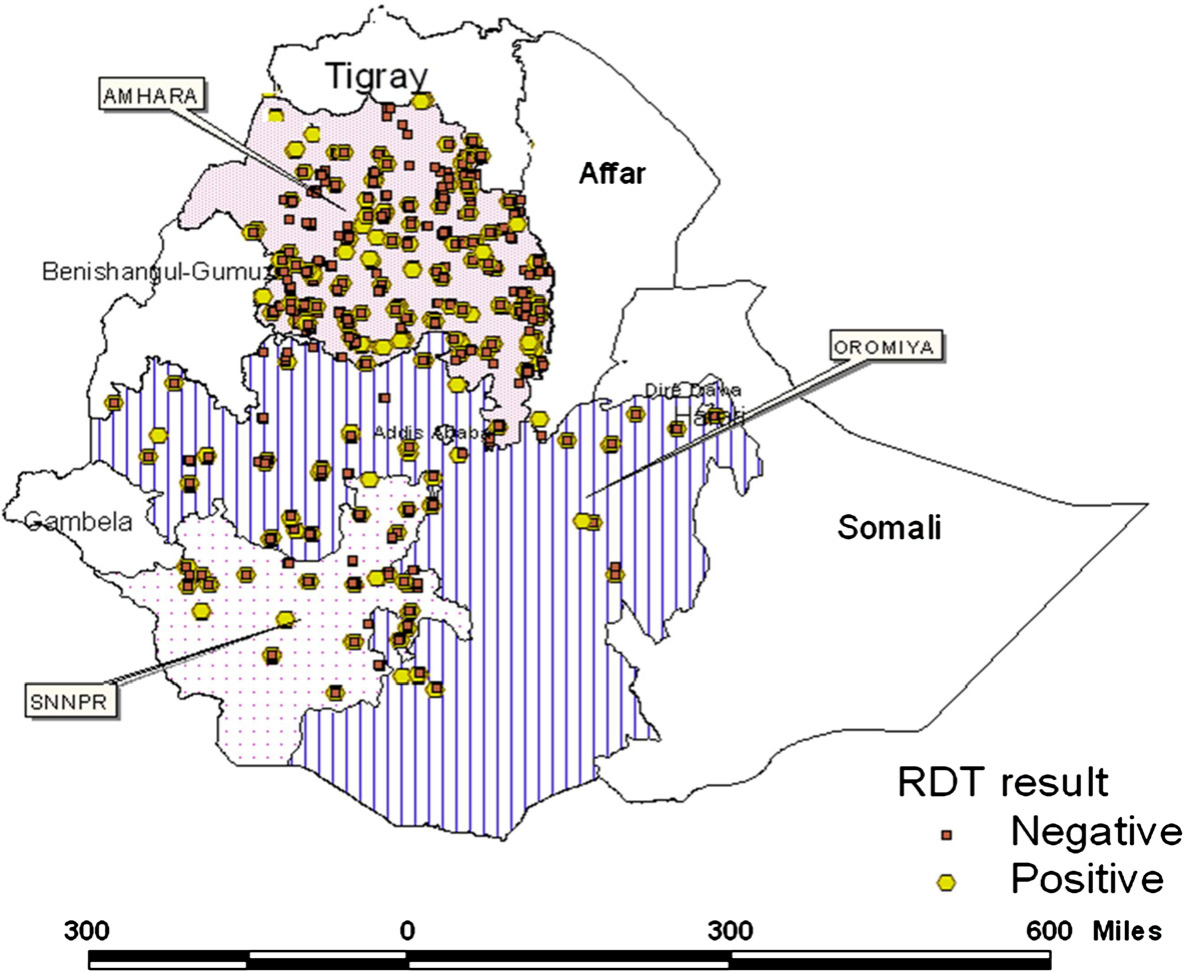
Classical and robust semivariogram for malaria prevalence.

Figure 5 represents different semivariogram estimators using classical and robust estimators. The classical estimator was suggested by Matheron in 1963 [42]. The classical estimator can be calculated by

$$\hat{Y}\left(h\right)=\frac{1}{\left|N\left(h\right)\right|}\sum_{N\left(h\right)}{\left(Z\left(s_{i}\right)-Z\left(s_{j}\right)\right)}^{2},$$

where (s_i_) is the anscombe residual,

$$N\left(h\right)=\left\{\left(s_{i}-s_{j}\right)\right):\left|s_{i}\right)-\left(s_{j}\right|=h\pm \left(\in \right\}\text{and}\left|N\left(h\right)\right|$$

is its cardinality. But, the classical estimator is sensitive to outliers. For this reason a robust estimator was proposed by Cressie and Hawkins in 1980 [43]. Among the different types of isotropic covariograms given above, Gaussian type was selected. Thus as discussed earlier, the best spatial covariance structure from all possible types was found to be the SP(GAU) (Gaussian) covariance structure. Therefore, the Gaussian type of the variogram was used to perform variogram analysis. The figure (Figure 3) shows first a slow, then a rapid rise from the origin. Therefore, the shape of the graph suggests a Gaussian type form which is given by

$$y\left(t\right)=c_{0}+c_{1}\left[1-\exp\left(-\frac{t^{2}}{R^{2}}\right)\right].$$

In general, from Figure 3, it is possible to distinguish three main features. The first one is the Y-axis well above zero, indicating the possible presence of a nugget effect. Moreover, the shapes of the semivariogram up through distances in the low 40s have roughly the shape of a spherical covariance model. Besides these, the semivariogram values are extremely high for the largest distances.

Tables 1 and 2 presents the significant effects for the model which incorporate spatial variability using SP (GAU) (Gaussian) covariant structure. Among all significant effects namely family size, altitude, toilet facilities, availability of radio and television, number of rooms per person, main material of the room's wall, spraying of anti- mosquito, use of mosquito nets and number of nets per person, were not involved in the interaction effects. The significant two-way and three-way interaction effects were found to be main source of drinking water and main material of the room's roof; time to collect water and main material of the room's floor; gender and main source of water; gender and main Material of the room's floor; age, gender and main source of drinking water; and age, gender and Availability of electricity. Based on these results for a unit increase in family size, the odds of positive rapid diagnosis test increases by 2.34% (OR = 1.0234, P-value < 0.0001). Furthermore, for a unit increase in altitude, the odds of positive rapid diagnosis test decreases by 1.4% (OR = 0.996, P - value <0.0001). With reference to individuals with no toilet facilities, the odds of a positive malaria rapid diagnosis test is lower for those individuals using a flushing toilet to those who have septic tanks (OR = 0.399, P - value <0.0001) or pit latrine slabs (OR = 0.644, P - value <0.0001). Moreover, for a unit increase in the number of total rooms, the odds of malaria diagnosis test for an individual decreased by 37.07% (OR = 0.629, P - value <0.0001). Similarly, with a unit increase in the number of nets in the house, the odds of rapid diagnosis test of malaria for individuals decreased by 60.7% (OR = 0.392, P - value <0.0001). Furthermore, for a unit increase in the number of rooms in the household sprayed with anti- mosquito, the odds of a positive malaria diagnosis test decreased by 53.3% (OR = 0.467, P - value <0.0001).

**Table 1:** **Socio**-**economic**, **demographic and geographic of effects on malaria RDT test for main effects**

**Parameters**		**Estimate**	**OR**	**SE**	**P -value**
Intercept		−0.2460	0.7819	5.8100	0.9995
Age		0.0209	1.0212	0.0503	0.6772
Gender (ref. male)					
	Female	−2.5463	0.0784	3.0804	0.4084
Family size		0.02311	1.0234	0.0527	<.0001
Region (ref. SNNP)					
	Amhara	−0.6896	0.5018	0.4502	0.1256
	Oromiya	−0.837	0.4330	0.5796	0.1487
Altitude		−0.0037	0.9963	0.0001	<.0001
Main source of drinking water (ref. protected water)					
	Tap water	−0.5557	0.5737	0.722	<.0001
	Unprotected water	0.6372	1.8912	0.6871	0.005
Time to collect water (ref. > 90 minutes)					
	< 30 minutes	−0.7829	0.4571	0.252	0.0019
	between 30 to 40 minutes	−0.603	0.5472	1.2666	0.6341
	between 40–90 minutes	−4.0189	0.0180	2.8957	0.1652
Toilet facility (Ref. No facility)					
	Pit latrine	−0.4403	0.6438	0.6433	<.0001
	Toilet with flush	−0.9177	0.3994	0.6413	<.0001
Availability of electricity (ref. no)					
	Yes	−3.1219	0.0441	1.0961	0.0044
Availability of television (ref. no)					
	Yes	0.6991	2.0119	0.2121	0.001
Availability of radio (ref. no)					
	Yes	−0.6991	0.4970	0.2121	0.001
Number of rooms/person		−0.4631	0.6293	0.0688	<.0001
Main material of room's wall (ref. cement block)					
	Mud block/wood	−4.1691	0.0155	1.2646	0.038
	Corrugated metal	−3.1196	0.0442	1.2576	0.004
Main material of room's roof (ref. corrugate)					
	Thatch	1.5031	4.4956	1.6732	0.005
	Stick and mud	0.454	1.5746	0.6726	0.0058
Main material of room's floor (ref. earth/Local dung plaster)					
	Wood	−1.1407	0.3196	0.803	0.004
	Cement	−0.9273	0.3956	0.114	0.028
Anti- mosquito spraying					
	No	1.237	3.4453	0.1734	<.0001
Use of mosquito nets (ref. no)					
	Yes	−0.8741	0.4172	0.1541	<.0001
Number of months room sprayed		−0.7626	0.4665	0.1274	<.0001
Number of nets/person		−0.9349	0.3926	0.0977	<.0001

**Table 2:** **Socio**-**economic**, **demographic and geographic of effects on malaria RDT test for interaction effects**

**Parameters**	**Estimate**	**OR**	**SE**	**P-value**
Gender and main source of drinking water (ref. Male & protected water)				
Female and tap water	−2.747	0.064	0.861	0.001
Female and unprotected water	1.224	3.402	1.064	0.250
Gender and material of room's floor (ref. Male and earth/Local dung plaster)				
Female and cement	−0.839	0.432	0.571	<.0001
Female and wood	0.762	2.143	0.387	<.0001
Age, gender and main source of drinking water (ref. Male & protected water)				
Female and tap water	−0.045	0.956	0.000	<.0001
Female and unprotected water	0.042	1.043	0.000	<.0001
Age, gender and availability of electricity (ref. Male & yes)				
Female and no	0.066	1.068	0.000	<.0001

### Interaction effects

**Figure 6:**
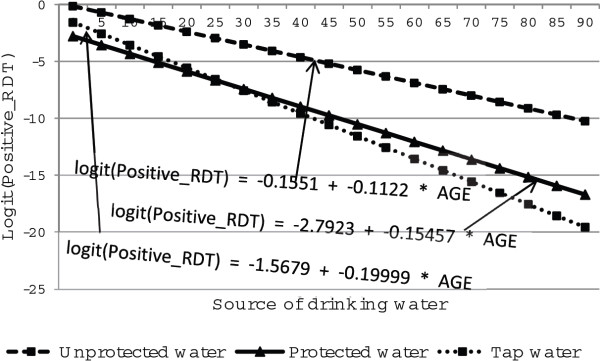
Log odds associated with rapid diagnosis test and age for male respondents with source of drinking water.

**Figure 7:**
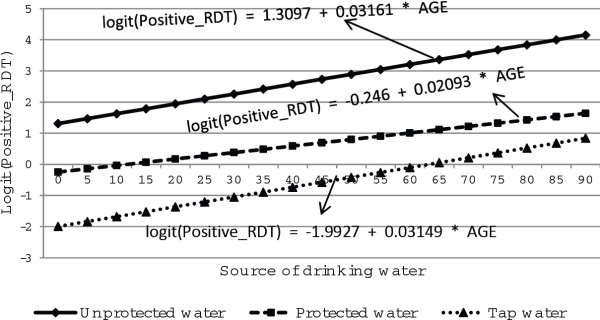
Log odds associated with rapid diagnosis test and age for female respondents with source of drinking water.

Figures 6 and 7 show the distribution of malaria rapid diagnosis test against age, main source of drinking water for both males and females respectively. As age increased, positive malaria diagnosis was less likely for males than females who were using protected, unprotected and tap water for drinking. Furthermore, as age of respondents increased, malaria rapid diagnosis test was less likely to be positive for individuals who use tap water for drinking for males and for females. More specifically, positive malaria diagnosis rate increases with age for females whereas it decreases as age increases for males (Figures 6 and 7). The figures further show that the gap in the rapid diagnosis test between respondents with unprotected, protected and tap water widens with increasing age.

**Figure 8:**
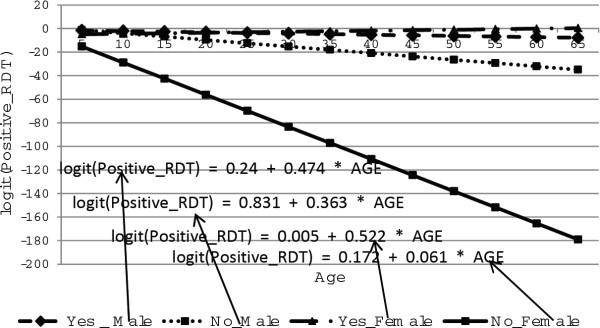
Log odds associated with rapid diagnosis test with age for male and female respondents with availability of electricity.

The relationship between age, gender and availability of electricity is presented in Figure 8. As the figure indicates, positive malaria rapid diagnosis test decreases as age increases for both male and female respondents, whether or not they have access to electricity, except for females who responded to having electricity. However, the rate of decrease was not the same for males and females after controlling for other covariates in the model.

**Figure 9:**
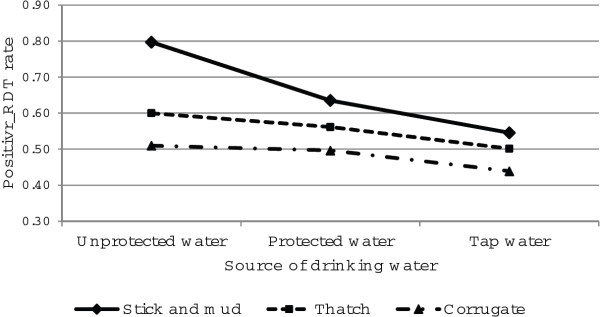
**Log odds associated with rapid diagnosis test and source of drinking water with material of the room's roof.**

Interaction effects between the main source of water and the main material used for the room’s roof is presented in Figure 9. From the figure, it is clearly seen that positive rapid diagnosis of malaria was significantly higher for households with a stick and mud roof followed by thatch and lastly a corrugated iron roof. This occurred with respondents who reported using tap water as well as protected and unprotected water for drinking (Figure 9). Furthermore, there was a significant difference in rapid diagnosis test between tap, protected and unprotected sources of drinking water for those who reported having thatch and stick and mud roofs. It is also shown that for corrugated iron roofs, the positive rapid diagnosis test was significantly lower for respondents who reported using tap water for drinking than for those who used protected and unprotected water for drinking.

**Figure 10:**
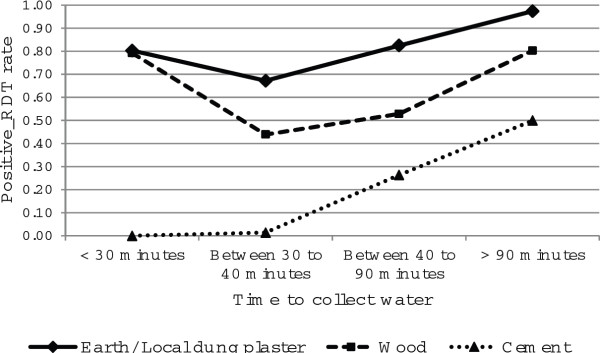
**Log odds associated with rapid diagnosis test and time to collect water with material of the room's floor.**

The other significant two-way interaction effect was between the time taken to collect water and the main flooring material (Table 2). This result is presented graphically in Figure 10. A positive rapid diagnosis test was significantly higher in those rooms with earth and local dung plaster floors than for those with cement and wooden floors, for respondents who took < 30 minutes and >90 minutes to collect water. But, for respondents who took less than 30 minutes to collect water but had a cement floor, the positive rapid diagnosis was low. Furthermore, with respondents who took between 30 to 40 minutes to collect water, there was a lower positive rapid diagnosis test for those with earth and local dung plaster floors compared to wooden floors.

**Figure 11:**
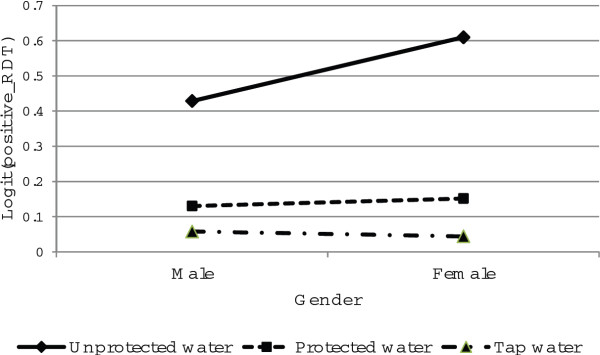
Log odds associated with rapid diagnosis test and main source of drinking water with gender.

The relationship between the main source of drinking water and gender is presented in Figure 11. As the figure indicates, a positive rapid diagnosis test was significantly higher for female respondents than for male respondents who reported using unprotected water. There was however, no significant difference in a positive rapid diagnosis test between females and males who reported using protected and tap water for drinking.

**Figure 12:**
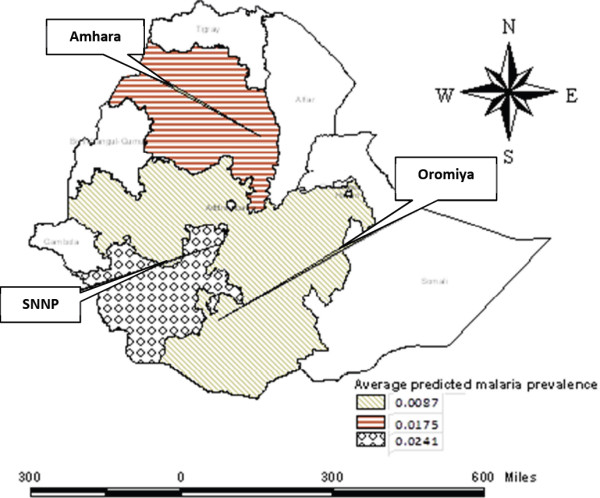
Predicted average spatial effects from the malaria prevalence model.

**Figure 13:**
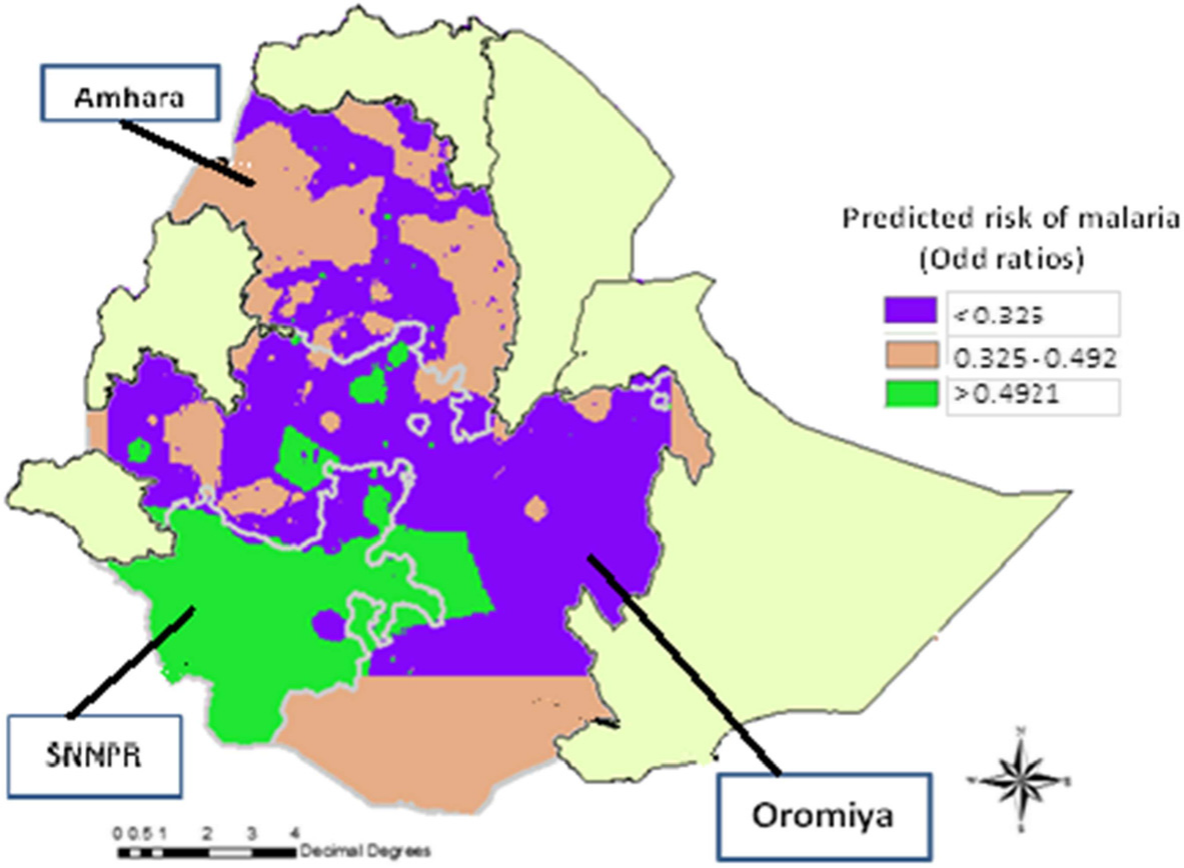
Predicted spatial effects from the malaria prevalence model.

The spatial model which is described above was used to produce a map of predicted prevalence of positive diagnosis malaria incidence rates for Amhara, Oromiya and SNNP regions of Ethiopia. When there is spatial data, the basic concern is the potential for spatial correlation in the observations. These spatial correlations could lead to incorrect estimates (estimates with underestimated standard errors). Spatial clustering of disease is almost to be expected since human populations generally live in spatial clusters rather than in a random distribution of space. An infectious disease that is highly associated with socio-economic, demographic and geographic factors is likely to be spatially clustered. This spatial clustering can occur even if the population distribution is not clustered. The model derived in this study explains some of the spatial patterns of the prevalence of malaria. The predicted prevalence of malaria is given in Figures 12 and 13. The prediction map (Figures 12 and 13) shows that the socio-economic, geographic and demographic factors are closely associated with the risk of malaria, mostly in the SNNP region followed by the Amhara and Oromiya regions. As can be seen from the map, the risk of transmission of malaria is of a moderately high intensity in almost all parts of the SNNP region. But, for the Oromiya region, the majority of households experience a lesser prevalence of malaria. Furthermore, from the map it can be seen that there is a high predicted value for the prevalence of malaria around the borders. This could be caused by cross-border migration of infected persons and the proximity of uncontrolled areas across the border, which may further add to the intensity of transmission in border areas.

## Discussion

The first priority in the acute stage of a malaria epidemic is prompt and effective diagnosis and treatment. Having well-planned and timely vector control can significantly contribute to a reduction in the risk of infection and consequently in saving lives. Vector control must be proactive and should be implemented at an early stage of epidemic development. Timing depends on effective early warning and early detection. Because of this, the government of Ethiopia has developed strategies related to human resource development, monitoring, and evaluation to control malaria and reduce the hardship it causes. Based on this strategy, the main objective of the government is to make those areas with historically low malaria transmission, malaria free and a near zero malaria transmission in the remaining malarious areas of the country [44]. Based on some studies which were conducted previously, malaria was regarded as a disease of the poor or a disease of poverty [45]. Looking at the global distribution of malaria in the world suggested that the concentration of the disease is in the world’s poorest continents and countries. Accurate information on the distribution of malaria in epidemic-prone areas on the ground permits interventions to be targeted towards the transmission and high-risk locations and households. Such targeting greatly increases the effectiveness of control measures but the inadvertent exclusion of these locations causes potentially effective control measures to fail. The computerized mapping and management of location data assists the targeting of interventions against malaria at the focal and household levels, leading to improved efficacy and cost-effectiveness of control.

As the distribution of malaria infection suggests, it is important to understand the relationship between malaria and poverty. This relationship is important to enable the design of coherent and effective policies and tools to tackle the problem [46,47]. As is already known, poverty is related to socio-economic factors. Therefore, it is important to identify those factors which are also related to the risk of malaria. Based on these facts, the findings from the current study show that the following socio-economic factors are related to the risk of malaria: main source of drinking water, time taken to collect water, toilet facilities, availability of radio, total number of rooms per person, main material of the room’s walls, main material of the room's roof, main material of the room's floor, spraying of anti- mosquito, use of mosquito nets, total number of persons per net. Besides socio-economic factors, there are demographic and geographic factors which also have an effect on the risk of malaria. These include gender, age and family size. In addition to the main effects there were interactional effects between the socio-economic, demographic and geographic factors which also influenced the risk of malaria. Most notable of these were the interaction between main source of drinking water and main material of the room's roof, time taken to collect water and main material of the room's floor, age and gender, gender and availability of electricity, gender and main material of the room's floor, age, gender and main source of drinking water; and age, gender and availability of electricity.

Spatially correlated data cannot be regarded as independent observations. Therefore, ignoring the spatial variability might lead to an inaccurate estimation of parameters. Accordingly, unlike Ayele, et al. (2012), the spatial correlation structure was considered and the significance of the variables was checked and predictions of the malaria risk levels for the sampled areas were produced. A useful way of providing up to date information is in the use of GIS-based management systems. This method helps to address effective malaria vector control and management. Therefore, the spatial distribution of malaria incidence was one of the points which were important for such GIS studies.

Spatial clustering of malaria is almost predictable as human populations generally live in spatial clusters rather than in random distributions of space. Disease which is highly correlated to socio-economic variables is likely to be spatially clustered. Therefore, the model explains some of the spatial patterns of malaria risk for Amhara, Oromiya and SNNP regions of Ethiopia. Moran’s and Geary's C tests were used to test for randomness [37-41]. The interest was to test if the spatial distribution of feature values is the result of random spatial processes. However, the test favors that the spatial distribution of feature values is not the result of random spatial processes. Moreover, the spatial distribution of high values and low values in the dataset is more spatially dispersed than would be expected. A dispersed spatial pattern often reflects some type of competitive process, i.e. a feature with a high value repels other features with high values; similarly, a feature with a low value repels other features with low values.

Malaria distribution is mainly related to the rainy seasons in Ethiopia. Therefore, understanding the nature of the Ethiopian climate is important. According to the Ethiopian National Meteorological Services Agency (NMSA), climates in Ethiopia can be divided into four climatic zones based on the pattern of rainfall. There are: the two-season type (the western half of Ethiopia) which is divided into district wet and dry seasons; bi-two season type (the south and southern of Ethiopia) is characterized by double wet seasons that occur between March to May and September to November with two dry seasons in between; the undefined season (dry northern part of the Ethiopian Rift Valley) mostly has irregular rainfall between July and February without any defined season; and the three-season type (central and south western Ethiopia). The average annual rainfall in the highlands of Ethiopia is above 1000 mm a year and it rises to 2000 mm and 3000 mm in the wet south western parts of Ethiopia. Therefore, the three regions have almost similar rainy months. Including the climate information into the analysis is important [48]. Since the climatic information is not included in the baseline household cluster malaria survey, this information will be included for future study.

Therefore, the results of this study provide evidence on the spatial distribution of socio-economic, demographic and geographic risk factors in the occurrence of malaria. This forms the basis for this research. Therefore, the utilization of socio-economic, demographic and geographic data on malaria rapid diagnosis test, including the information on the spatial variability, clarifies the effects of these factors. From the study it was observed that residents living in the SNNP region were found to be more at risk of malaria than those living in Amhara and Oromiya regions. Similarly, houses which were treated with anti- mosquito spray were less likely to be affected by malaria. However, a major challenge in the control of malarial infection was found to be in the type of toilet facilities available in the household. From the results, it was observed that individuals living in households which had no toilet facilities were more likely to be positive for malaria diagnosis tests. Furthermore, positive malaria diagnosis rates decreased with age and the risk of malaria increased per unit increase in family size. Generally, malaria parasite prevalence differed between age and gender, with the highest prevalence occurring in children and females.

From the findings of this study, it can be suggested that having toilet facilities, access to clean drinking water and the use of electricity offers a greater chance of knowing whether or not an individual in the household is at risk of malaria or not. In addition to this, using mosquito nets and spraying anti- mosquito treatment on the walls of the house were also found to be a way of reducing the risk of malaria. Similarly, having a cement floor and corrugated iron roof was found to be one means of reducing the risk of malaria. Based on the findings, different types of housing materials have an influence on the risk of malarial transmission with those houses constructed of poor quality materials having an increased risk. Moreover, the presence of particular structural features, such as bricks, that may limit contact with the mosquito vector, also helps to reduce infection. The risk of malaria therefore, is higher for households in a lower socio-economic bracket than for others who may enjoy a higher status and who are able to afford to take measures to reduce the risk of transmission. Therefore, with the correct use of mosquito nets, anti- mosquito spraying and other preventative measures, like having more rooms in a house, the incidence of malaria could be decreased. In addition to this, the study also suggests that the poor are less likely to use these preventative measures to effectively counteract the spread of malaria. To provide clean drinking water, proper hygiene and maintaining the good condition of a house is essential in controlling the transmission of malaria. With other control measures, including creating awareness about the use of mosquito nets, anti- mosquito sprays and malaria transmission, the number of malaria cases can be reduced. Furthermore, spatial statistics studies significantly contribute to the understanding of the distribution of malarial infections. The use of spatial statistics analysis is effective in monitoring and identifying high-rate malaria affected regions and helpful when implementing preventative measures. Finally, studies incorporating spatial variability are necessary for devising the most appropriate methodology for remedial action to reduce the risk of malaria.

## Ethical clearance

The ethical protocol received approval from the Emory University Institutional Review Board (IRB 1816) and Amhara, Oromiya and SNNPR regional health bureaux. Informed consent was sought in accordance with the tenets of the declaration of Helsinki.

## Competing interests

The authors declare that they have no competing interests.

## Authors’ contributions

DGA acquired the data, performed the analysis and drafted the manuscript. TTZ and HGM designed the research. All authors discussed the results and implications and commented on the manuscript at all stages. All authors contributed extensively to the work presented in this paper. All authors read and approved the final manuscript.
